# User Perceptions of ¡Protéjase!: An Intervention Designed to Increase Protective Equipment Use Among Mexican Immigrant and Mexican American Farmworkers

**DOI:** 10.2196/mhealth.4455

**Published:** 2016-04-11

**Authors:** Shedra A Snipes, Francisco A Montiel-Ishino, Joshua M Smyth, Dennis J Murphy, Patricia Y Miranda, Lisa A Davis

**Affiliations:** ^1^ Penn State University Department of Biobehavioral Health University Park, PA United States; ^2^ Penn State University Department of Agricultural and Biological Engineering University Park, PA United States; ^3^ Penn State University Department of Health Policy University Park, PA United States; ^4^ Pennsylvania Office of Rural Health University Park, PA United States

**Keywords:** mHealth, Hispanic, migrant worker, intervention study, pesticides, occupational safety

## Abstract

**Background:**

Farmworkers’ exposures to pesticides are reduced when they wear personal protective equipment (PPE), and mobile health (mHealth) platforms can potentially deliver information to farmworkers to help promote PPE use. However, little is known about the feasibility of using mHealth platforms to promote farmworkers’ use of PPE.

**Objective:**

The objective of the study was to describe the development and feasibility-testing of Protect Yourself! (¡Protéjase!), an intervention designed to increase PPE use. As the vast majority of farmworkers in the United States are from Mexico, we examined the intervention in a primarily Mexican-origin farmworker population.

**Methods:**

¡Protéjase was developed in several steps. First, we performed ethnographic observations to understand what prevents PPE use. Next, we developed program components that met the challenges uncovered in the ethnographic observations, seeking direct feedback from farmworkers on each component. Feasibility was assessed using surveys and focus groups. Material was provided in Spanish or English at the preference of the participant. Finally, we pilot tested each component of the intervention, including: (1) PPE that was provided to each worker for their personal use during the intervention trial, and (2) delivery of an application-based tool that promoted the use of PPE through daily individualized messaging.

**Results:**

55 farmworkers enrolled in the study, but only 41 of 55 (75%) completed the entire pilot intervention trial. Results focus on the evaluation of the intervention, and include only those who completed the entire trial. Among farmworkers who completed the entire intervention trial, all but two farmworkers were born in Mexico and were Spanish speaking. Still, all study participants self-identified as Mexican or Mexican-American. When asked what changes were needed in the intervention’s messaging or delivery to increase user satisfaction, 22 out of 41 participants (54%) felt that no changes were needed. However, 16 of 41 participants (39%) suggested small changes to messaging (eg, refer to long pants as pants only) to improve their understanding of the messages. Finally, a small number (3 of 41 participants, 7%) felt that messages were difficult to read, primarily due to low literacy.

**Conclusions:**

The ¡Protéjase! mHealth program demonstrated very good feasibility, satisfaction, and acceptance; potential improvements (eg, small modifications in messaging to increase farmworkers’ use) were noted. Overall, the PPE provided to workers as well as the mHealth platform were both perceived as useful for promoting PPE use.

## Introduction

The use of personal protective equipment (PPE) significantly reduces workers’ exposures to pesticides [1-5]. Further, PPE is recommended by the Environmental Protection Agency as the primary way to protect farmworkers who may be exposed to pesticides as they work [6]. However, exposure to pesticides among farmworkers is not equal. Over 80% of the agricultural workforce is of Mexican origin [7], and recent research indicates that farmworkers who identify as Latino have significantly greater exposures to pesticides [8]. It is critical, then, to develop new venues for interventions that can help promote pesticide safety for Mexican and Latino farmworkers.

Intervention strategies such as the use of mHealth (broadly defined as the use of mobile and wireless devices to improve health, health services, and health research) present a unique and viable intervention platform to improve PPE use. Moreover, there is preliminary evidence that Mexican and Latino farmworkers are likely to have mobile phones and are willing to receive health information in a mHealth format [9-11]. However, utilizing a mHealth approach for pesticide safety among a Mexican farmworker population has not yet been explored. As such, we developed and implemented a mHealth intervention named Protect Yourself! (¡Protéjase!), which was both broadly (eg, language, culturally appropriate) and dynamically (daily risk profiles) tailored to promote PPE use among Mexican farmworkers. Our efforts build on the growing use of mHealth as an approach to capture daily data to implement messaging based on individual-level characteristics, such as changes in daily risk factors [12]. Specifically, our objective was to pilot test each component of the intervention, including: (1) provision of PPE (long-sleeved shirts, gloves, and safety glasses) that was provided to each worker for their personal use during the intervention trial and (2) delivery of an application (app)-based tool that promoted the use of PPE through daily individualized messaging.

## Methods

### The Steps of ¡Protéjase! Intervention

#### Step 1

**Figure 1:**
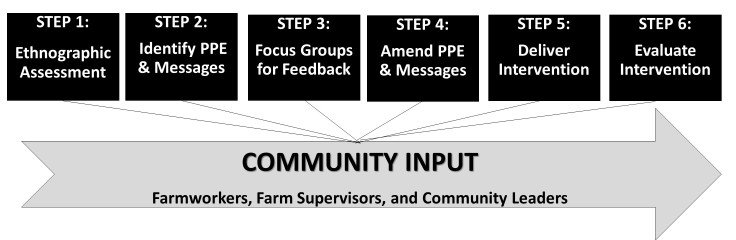
¡Protéjase! development steps. Personal protective equipment: PPE.

The ¡Protéjase! intervention was developed in six steps (Figure 1 shows this). Step 1 entailed a series of ethnographic assessments performed by the primary investigator. The overall goal of ethnographic assessments was to better understand why workers did not wear their PPE, or removed it (eg, because it interrupted or slowed their work). Data for the ethnographic components were collected during 3 periods: 3 months of spring evaluation (April 2009 to June 2009), 10 months of winter and spring evaluation (October 2009 to July 2010), and 3 months of summer evaluation (June 2011 to August 2011). The participant samples were 32, 30, and 35 respectively. Participants for the ethnographic studies were all recruited through parent meetings at Teaching and Mentoring Communities, Inc (TMC), a nonprofit organization with a Migrant and Seasonal Head Start program that serves approximately 8000 children of parents who work in agriculture. TMC is located in the Lower Rio Grande region of Texas where a significant portion of residents are farmworkers [13].

Ethnographic observations of PPE use behaviors typically took between 6-9 hours, and occurred 5-7 days per week for a total of 3387 observational hours. Additional time in the field included transcribing notes, which were captured using a digital voice recorder and written in a notebook using a shorthand system that allowed the investigator to quickly record observations. Also, 74 interviews were conducted that inquired about reasons behind PPE use/nonuse, ranging from 30 minutes to 3 hours each. Finally, between 2-5 hours of every observation day was used writing field diary notes, coding notes, listening to voice recordings of interviews, and keeping an active field diary. The relevant Institutional Review Boards (2009-2010 by MD Anderson Cancer Center; 2011 by the Pennsylvania State University) approved all work for the ethnographic studies.

The ethnography produced numerous examples of why farmworkers removed their PPE, or did not put it on at all. For example, previous work has described that farmworkers’ beliefs about individual vulnerability and machismo may influence pesticide exposures [14-16]. Building upon that literature, our observations showed that someone who self-perceives their body as “delicate” or “weak” in their organismo (body-type) might more readily wear their PPE. In fact, workers described as delicados (persons with a delicate body type) were encouraged by their family or coworkers to put on PPE even if it slowed them down. On the other hand, we found that PPE is strongly perceived as a barrier to efficient work, and is seen as preventing most workers from achieving important harvest quotas (which, when not met, may result in loss of pay or employment). Thus, workers who had a stronger “organismo" were less likely to wear PPE because their body type was perceived as strong enough to forgo PPE use, and because PPE decreased their work efficiency.

#### Step 2

Next, in step 2, we began to develop program components that met the challenges uncovered in the ethnographic observations. First, we sought PPE that would be perceived as practical enough to not impede productivity. Further, we aimed to establish messages designed to address key barriers for the use of PPE, thus enhancing motivation for and execution of or execution of PPE. To do this, we first identified multiple options of each type of PPE (ie, long sleeved shirts, safety glasses, and gloves) that could be worn comfortably throughout the workday. PPE was located by contacting occupational equipment companies whose products were a potential match for the criteria of functional wear without impeding work productivity. Criterion factors included fabric with heat cooling technology, nonfog lenses, and gloves that promoted maximum dexterity. In total, we were able to identify 22 options, which included 4 types of shirts, 10 types of gloves, and 8 types of safety glasses. Next, culturally appropriate messages were drafted. To create messages, we used a matrix-based guiding framework from Intervention Mapping [17]. Message development started with a matrix that placed behavioral objectives in rows, which included goals such as putting on a long-sleeved shirt and keeping the long-sleeved shirt on all day. Determinants that influenced the goals were placed in columns, and included perceived barriers, perceived benefits, self-efficacy, knowledge, and attitude (among others). In a row-by-row fashion, we created multiple corresponding messages for each cell in the matrix attempting to minimize repetitiveness (resulting in a total of 309 messages). Also, the wording of each message considered that delivery was to take place in an individually tailored manner, and in direct response to their actual daily behavior. For example, a sample message was: “We know - sometimes it’s hard to wear protective clothing. Today you did not wear your gloves because they were uncomfortable. However, we want you to know that pesticides can be harmful when you are exposed to them. In order to protect yourself, wear the appropriate protective clothing every day”.

#### Step 3

In step 3, we conducted 5 focus groups among 50 adult farmworkers (10 farmworkers per focus group) to gain their opinions on both the PPE and draft messages developed in step 2. Focus groups were approved by the Institutional Review Board at Penn State University, and held at the TMC facilities. We recruited 50 farmworkers by attending parent meetings at TMC. Among the 50 focus group participants, they provided feedback on types of PPE, which were evaluated by farmworkers for both comfort and productivity (uncomfortable/slows productivity, comfortable/slows productivity, helps productivity/uncomfortable, helps productivity/comfortable). Additionally, intervention messages were discussed and evaluated, and then group-ranked according to their potential impact to motivate increased PPE use.

#### Step 4

In step 4, we reviewed farmworkers’ feedback on PPE and messages. First, based upon farmworkers’ suggestions, PPE was narrowed from 22 to 7 types that farmworkers perceived could be feasibly worn throughout the workday (1 shirt, 3 types of gloves, and 2 types of safety glasses). We kept PPE according to farmworker’s rankings of highest level of protection in conjunction with their most pragmatic needs (ie, comfort and productivity). Representative quotes of farmworker’s perceptions of highly evaluated PPE are available in Table 1. Next, the draft messages were evaluated. Based on farmworker’s feedback, messages were narrowed from 309 messages to 209 messages. Most messages were removed because farmworkers viewed them as too lengthy, or not convincing enough to motivate PPE use. A small number of messages were removed because they repeated the overall goal of other messages. The top-rated PPE and edited messages were integrated into the intervention design.

**Table 1:** Representative quotes regarding perceptions of highly evaluated PPE.

PPE	Farmworker quotes
Gloves	"These gloves fit better and are more comfortable, they would be good for jobs that require handling small fruit picking." "These gloves are perfect for working with tasks that require good grip while handling sharp plants or knives."
Long sleeve shirt	"This shirt is good for work because it protects you all the way to the wrist, it looks comfortable, it looks like a dress shirt, you can put your pen, and small tools in the pockets."
Glasses	"These glasses are great for when we bend down. You can use them with the straps too so they don’t fall off while you are working." "These glasses are good for when you are driving a machine.”

#### Step 5

Next, in step 5, we pilot tested the intervention in a new sample of 55 farmworkers (no farmworkers from the focus groups participated in the pilot delivery trial). The Institutional Review Board at Penn State University approved all pilot study components. Program participants were recruited from TMC through parent meetings, and written informed consent was obtained in Spanish or English, depending on participant preference. In order to ensure consistency in the function of the pilot mHealth app, and to standardize presentation of materials (eg, screen size, power, maintenance, etc), farmworkers were provided with mobile phones, instruction on basic phone use (eg, how to turn the phones on-off), and trouble-shooting of technical issues (ie, how to deal with frozen screens) prior to starting the pilot intervention. Participants were then taught how to use the ¡Protejase! mHealth app, including how to respond to daily surveys. Farmworkers were also provided the optimized PPE (long-sleeved shirts, gloves, and safety glasses). Additional training was provided to farmworkers on the correct use of PPE with tips for increased comfort and wear while working in the fields. Each training session took approximately 45 minutes. Finally, a toll free number with 24-hour accessibility to project staff was provided to participants to call if they had questions or issues with mobile phones or PPE for the duration of the study. Participants were given a US $15 participant Wal-Mart gift card and were able to keep the PPE after the pilot study concluded.

**Figure 2:**
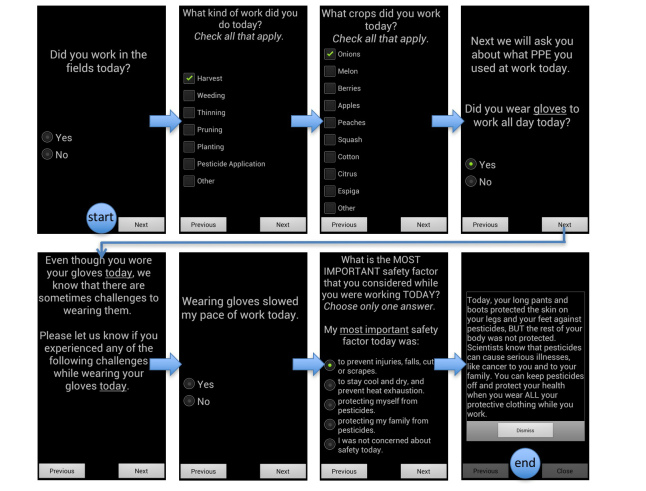
Dynamically tailored ÃƒÆ’Ã†â€™ÃƒÂ¢Ã¢â€šÂ¬Ã…Â¡ÃƒÆ’Ã¢â‚¬Å¡Ãƒâ€šÃ‚Â¡ProtÃƒÆ’Ã†â€™Ãƒâ€ Ã¢â‚¬â„¢ÃƒÆ’Ã¢â‚¬Å¡Ãƒâ€šÃ‚Â©jase! daily survey example.

To deliver individually and dynamically tailored messages to each person in the pilot study, a short daily survey was delivered via the ¡Protejase! mHealth app at the end of each work day. The survey assessed information for that current day, including work hours, the type of work performed, the type of crop harvested, pesticide application, PPE use, reasons why PPE was worn (or not, tailored to responses), and farmworkers’ perceptions of work safety. After completing the survey, farmworkers received an individually tailored message based on their responses to the survey for that day. As alluded to earlier, messages were designed to be responsive to all possible combinations of PPE use scenarios, risk beliefs, and work tasks. To accomplish this without becoming repetitive (particularly in the context of repeated PPE failures in the same domain), the app contained 5 different messages per potential scenario to prevent the same message being delivered if participants engaged in the same behaviors for more than one day (messages could, however, repeat after 5 instances). As an example of message tailoring, if a participant indicated that protective gloves were not worn, they were prompted to provide the reason the PPE was not worn (eg, forgot to wear them, too hot, uncomfortable, slowed productivity, etc). We also assessed any perceived difficulties or negative consequences of wearing PPE, if worn. When participants indicated, for example, that they did wear gloves that day, they were prompted to “Please let us know if you experienced any of the following challenges while wearing your gloves today” and could indicate if glove use slowed productivity, was too hot, uncomfortable, etc. Answers to the completed survey were then assembled and matched to a motivational cue specifically tailored to remind the participant to wear the appropriate type of PPE (in this example, gloves) the following day. Motivational messages were provided on a daily basis, and included reminders to wear PPE, information about the health risks of pesticides, and helpful tips to use PPE effectively to remain safe at work (Figure 2 shows an example). The pilot test was implemented daily over a 30-day period.

#### Step 6

Finally, we evaluated the feasibility and preliminary effectiveness of ¡Protejase! in step 6. To evaluate the intervention, baseline and follow-up surveys were used to assess feasibility characteristics as well as change in PPE use in response to the intervention trial. Feasibility assessment included perceived barriers to PPE use, loss to follow-up, comprehension of mHealth messages, survey and message delivery time and frequency of messages, ease or difficulty of mobile phone use, battery life, and phone screen issues (such as freezing and font size). Additionally, all participants who completed the entire 30-day intervention participated in one focus group at the close of the intervention to provide open feedback on the intervention feasibility.

### The Analysis

For analyses, basic descriptive statistics assessed average percentages and ranges. Additionally, Pearson’s linear correlations and simple linear regression were used to understand relationships between barriers in mobile phone use and mHealth assessment since the intervention. Linear associations were analyzed controlling for potential influence of income, education, and mobile phone ownership as each could affect barriers to mobile phone use among participants. For qualitative analyses of the evaluation focus groups, data were coded by two independent coders and underwent thematic content analysis. Coding and analysis was iterative, and themes were derived by frequency of codes (most occurring concepts), repetition of perceptions across subjects and groups, and consensus of perception by farmworkers’ demographic attributes. To assess concepts across groups and attributes, codes were linked and explored for patterns based on demographic profiles to compare and identify common themes among participants.

## Results

### Intervention Enrollment

A total of 55 farmworkers were enrolled and began the intervention trial, with 41 (75%) completing the 30-day study. Evaluation of the intervention is based on the sample (n=41) that completed the trial and were available at follow-up. Participant characteristics at baseline and follow-up are included in Table 2.

#### Farmworker Mobile Phone Use

All participants identified as Mexican or Mexican-American. There was high preexisting mobile phone use in this population both in the enrollment sample, and in the sample that completed the entire intervention trial. Among farmworkers who owned a mobile phone, (n=14/34) 41% indicated using it on a regular basis for phone calls to-and-from family and friends.

**Table 2:** Demographic profile of ¡Protéjase! feasibility study participants at baseline (n=55) and follow-up (n=41).

		Baseline		Follow-up	
		n	%	n	%
**Language**					
	Spanish	52/55	95	39/41	95
	English	3/55	5	2/41	5
**Sex**					
	Male	31/55	56	23/41	56
	Female	24/55	44	18/41	44
**Place of birth**					
	Mexico	48/55	87	39/41	95
	United States	7/55	13	2/41	5
**Age**					
	< 20	3/55	5	1/41	2
	21–30	28/55	51	22/41	54
	31–40	14/55	26	11/41	27
	41 +	8/55	15	6/41	15
	Don’t know/refused	2/55	3	1/41	2
**Education completed**					
	< 8^th^ grade	24/55	44	17/41	42
	9^th^ -11^th^ grade	13/55	24	10/41	24
	12^th^ grade or GED	12/55	22	10/41	24
	Some college baccalaureate	3/55	5	2/41	5
	Don’t know	3/55	5	2/41	5
**Annual Income (US)**					
	< $10,000	28/55	51	21/41	51
	$10,000-$14,999	10/55	18	8/41	20
	$15,000-$24,999	5/55	9	3/41	7
	Don’t know	12/55	22	9/41	22
**Owned a mobile phone for personal use**					
	Yes	41/55	75	34/41	83
	No	13/55	24	6/41	15
	Don’t know	1/55	1	1/41	2
**Mobile phone use (among those who own a mobile phone)**					
	Always	10/41	24	9/34	26
	Sometimes	6/41	15	5/34	15
	Hardly	25/41	61	20/34	59

### Personal Protective Equipment Satisfaction and Acceptability

The overwhelming majority of participants were pleased with the PPE and mHealth messages. The long-sleeved shirts were described as “...good, [well] ventilated and fresh (...estaba bien, tenia ventilacion, estaba fresca)” because it “kept [them] dry and free from sweat (...mantenia seco...sin sudor)”. Safety glasses were seen as a welcome addition as they were provided in various tints, “were ventilated (estaban ventilados)” and did not “fog”. Gloves were generally liked because, in addition to protecting hands, they “limited rashes” among harvesters.

A small number of barriers were reported that limited practical PPE use. In the case of long-sleeved shirts, issues were only related to sizing. A participant reported “[their] shirt was very large [and] uncomfortable [because] the sleeves would get stuck and become bothersome (la camisa estaba muy larga...[y] era incomoda[...]se atoraba[...]y estorbaban las mangas)”. Other barriers to PPE use were crop specific to cilantro harvesting, which required the modification of gloves to allow bunching and tying of the crop. A participant expressed this sentiment noting, “gloves did not work well for cilantro...they only work well for certain tasks (los guantes no[...]sirvieron mucho...[son buenos para ciertas cosas])”. To deal with the practical issues of glove use with cilantro, participants often modified gloves by cutting off the tips on the thumbs and index and middle fingers, giving them the dexterity they needed to bunch and tie the crop in the field, while maintaining some (more limited) degree of protection. It should be noted that participants indicated that prior to the intervention, they did not wear gloves at all while harvesting cilantro, but wore them in a modified way after provision by the ¡Protéjase! program.

An additional barrier with the gloves was that they had high turnover dependent on the speed and output of the harvester. Participants also commented that they might need more than 2 pairs of each glove over the 30 day timeframe (the average observed turnover of gloves among all farmworkers was 2 weeks for each pair, suggesting our provision of 2 pairs was a roughly adequate timeframe for the length of the study period). Despite the barriers, participants expressed that PPE was beneficial and important overall.

### mHealth Satisfaction and Acceptability

The daily survey and PPE motivational messages were also well regarded, as indicated by one participant who stated: “the questions were easy (las preguntas estaban fáciles)” and “[my] favorite [part] was the messages ([mi parte] favorita fueron los mensajes)”. According to participants (see Table 3), the most useful messages were about the health effects of pesticides and risk to their family (collectively selected as most useful by 27/41; 66%) and tips and reminders on PPE use (13/41; 32%). When asked about possible message changes, many participants (22/41; 54%) liked all risk-reduction messages and felt that no changes were needed; (n=16/41) 39% liked the messages, but felt that some refinement (such as small changes in language) was needed to most effectively increase PPE use. As an example of a suggested change, participants found use of the term “long pants” confusing and suggested just referring to them as “pants” only. Finally, (3/41) 7% felt that messages were difficult to read, which appeared to primarily reflect low literacy levels of some participants. We also established in focus group discussions that some participants (7/41; 17%) had trouble navigating the mobile phones due to literacy issues. In those cases, five spouses/wives and two children helped participants complete the daily survey. Each of the seven participants who reported literacy issues in the focus groups said that they were able to successfully complete the survey each day in one sitting because of the help of a family member or spouse to read the daily survey and messages. See Table 3 for a detailed breakdown on mHealth message satisfaction.

### Barriers to Mobile Phone Use

The primary perceived barrier to the mHealth approach was difficulty with technical issues regarding the mobile phone (such as battery life or freezing of the phone’s screen). The majority of participants (30/41; 74%) indicated they had no barriers using the mobile phone and reported the questions as “being easy (estaban fáciles)”. Of the approximately (11/41) 27% of participants who reported barriers, these barriers appeared related to technological challenges in accessing the survey on the phone. Despite any such barriers, provision of daily surveys was quite good. Overall, 786/959 (81.9%) surveys were successfully completed in one attempt, an additional 148/959 (15.4%) surveys in two attempts, and a small fraction requiring three or more attempts (25/959; 2.6%).

Our data also show that the average length of time required to complete the daily survey was moderately associated with perceived barriers of mobile phone use [*r=* 0.355; *P*=.025], suggesting that participants who reported barriers were more likely to take longer to complete daily surveys. We note that both reported barriers and length of time to complete daily surveys were higher in farmworkers with less than 8^th^ grade education; even in this group, all completed surveys required 8 minutes or less. Across the entire sample available at follow-up, the average length of time to complete surveys was 5 minutes.

### Study Retention and Mobile Phone Loss

As noted, most participants, (41/55) 75%, were maintained throughout the 30-day intervention trial. We examined those lost to the study (n=14). There were two participants that initially self-reported as being over the age of 18, and subsequently reported that they were 17 years old; several were not able to complete follow-up surveys (n=5) because of travel to Mexico to visit sick family or friends; and some had lost their work (n=7), as migrant labor is seasonal and often unstable. Among those who lost work, the individuals did not complete the intervention given that the primary goal of the intervention evaluation stage was to examine the impact on PPE use at work, and we collected data on those participants who worked throughout the 30 day study period. Overall, attrition did not appear to be due to “actual” concerns with the intervention or mHealth procedures, but rather seemed largely driven by external factors. Participants were asked to return the study mobile phones at the conclusion of the intervention pilot, and phone loss was low; (50/55) 90% of phones were returned at study completion. Even among the 14 participants not successfully completing the intervention component, 9 (64%) managed to return the phone to study staff.

**Table 3:** mHealth message satisfaction of ¡Protéjase! feasibility study at follow-up (n=41).

		n	%
**What was the most useful type of information?**			
	Health effects about pesticides	18/41	44
	Risks for my family	9/41	22
	Reminders about wearing PPE	7/41	17
	Tips on how to use PPE	6/41	15
	Don’t know/not sure	1/41	2
**What impacts did the messages have on PPE use?**			
	A big increase in my use of PPE	17/41	41
	A small increase in my use of PPE	4/41	10
	No change in my use of PPE	9/41	22
	Decreased my use of PPE	4/41	10
	Don’t know/not sure	7/41	17
**What did you think of the messages you received?**			
	I liked the messages, no need for changes	22/41	54
	I liked the messages, but they need refining	5/41	12
	Some messages were easier to understand than others	9/41	22
	All messages were difficult to read/understand	3/41	7
	Don’t know/not sure	2/41	5
**What barriers did you encounter while using the mobile phone?**			
	None	30/41	73
	Difficulty in using/navigating the mobile phone to take survey	1/41	2
	Screen of the mobile phone froze	3/41	7
	Issues with battery (low battery)	6/41	15
	Fonts size made it difficult to read	1/41	2

## Discussion

### Principal Findings

¡Protéjase! was developed to increase PPE use in Mexican farmworkers through tailored prevention messages. Our evaluation showed that the program was viewed by workers as acceptable and appropriate to their cultural attitudes, and demonstrated very strong feasibility as an integrated intervention platform (PPE provision coupled with an individually and dynamically tailored mHealth motivational app). Satisfaction with the PPE component of the intervention was primarily linked to farmworkers’ consideration that the PPE was comfortable and would not (or minimally) negatively impact work productivity. Also, the individualized messages were perceived as most helpful when they communicated health risks, or placed messages in the context of how to protect the family.

Previous literature strongly suggests that farmworkers often perceive PPE as disruptive to work efficiency and, when this is the case, farmworkers are not inclined to wear it. For example, Quandt et al [18] report that almost half of 197 farmworkers did not wear safety glasses because they prevent workers from distinguishing between the leaf colors of plants during harvest. A more recent intervention by Strong et al [19] reported that although pesticide safety knowledge scores increased in response to an intervention, change in glove and safety glasses use were unchanged because of impractical PPE. Moreover, the literature also strongly recommends that the impractical function of PPE is a primary barrier that must be overcome to boost pesticide safety behaviors among farmworkers [20].

¡Protéjase! begins to respond to the challenge about PPE impracticality by providing farmworkers with PPE that the workers perceived as comfortable to wear, but that did not meaningfully slow their production. Moreover, farmworkers felt that having comfortable PPE increased their use of it. This is notable, as it is well established that PPE has the potential to significantly lower pesticide exposure levels [2-6]. If farmworkers are able to increase their PPE use through the provision of high quality PPE (and the receipt of appropriate risk messaging through a mHealth app or the receipt of appropriate risk messaging through a mHealth app, discussed below), it is likely that their exposures may also be reduced.

Additionally, farmworkers responded well to the messaging components of the study, and the majority of participants found the mHealth platform on provided mobile phones a viable option for their daily use. This may be due, at least in large part, to the substantial preexisting ownership of (and, presumably, familiarity and comfort with) mobile phones. This, coupled with previous research demonstrating that farmworkers have broad access to mobile phones [9], suggests that mHealth may hold tremendous potential as a platform for interventions for farmworkers. We did not, however, attempt to use participants’ own mobile phones in our study to avoid an array of logistical issues (eg, software and other procedural features need to work correctly across different phone operating systems and versions, various screen sizes, compatibility among various platforms, etc); rather, for this initial study, we provided all participants with phones for use during the study period. As such, future work should evaluate the use of personal phones in order to more readily be able to bring interventions to scale.

Finally, we established that farmworkers were willing and able to fully participate in mHealth programs to increase PPE use. Others have previously suggested that as technology becomes increasingly familiar to farmworkers, that mHealth approaches/services have tremendous potential to provide access to a wide range of their health care needs [9-11]. In the future, mHealth interventions like ¡Protéjase! may provide a framework that can be replicated and feasibly applied to a broad array of public health issues, ranging from health promotion to interventional behavior modification for chronic illness. In this regard, utilizing a mHealth approach to collect data from farmworkers as they go about their daily lives may be an important new area that strengthens existing work in that population by enhancing the accuracy and ecological validity of behavioral reports in safety and health research.

### Limitations

Although participants largely viewed our intervention as successful, there are areas for improvement. For example, our intervention language was created at a 5th grade level of education. Despite this, workers recommended tailoring language and making the intervention more accessible for low-literacy workers. National estimates suggest that farmworkers generally have low-levels of education [8] and most farmworkers in our study had less than a high school education. Although only (3/41) 7% of participants had issues with reading the messages, future versions of our program may be able to resolve literacy issues by using voice automation to improve its acceptability in low-literacy workers. Automated programs have proved largely successful in other mHealth studies for low-literacy farmworkers [10,11] and might improve the feasibility and implementation of our program. We also note that messages in future versions of our program might be extended to encompass additional safety domains. For example, to remind workers when they should wash and launder their PPE, as PPE is most effective when it is clean. Finally, we note that this study takes place in Texas only, and has a small sample size. Our findings may not be generalizable to all farmworkers.

### Conclusions

In summary, the use of an integrated intervention approach, coupling the provision of optimized PPE with a supportive mHealth app, to deliver ¡Protéjase! to farmworkers was well-received, and we see this approach as an innovative way to engage farmworkers for pesticide protection. Additionally, mHealth approaches like ¡Protéjase! might serve as model programs that could be altered to address other health issues in farmworker populations by dynamically tailoring messages to their specific daily needs. In this regard, the mHealth platform can be a useful design for integrating culturally appropriate health messaging and data collection for pesticide safety and health information delivery.

## Abbreviations

app: application
PPE: personal protective equipment
¡Protéjase!: Protect Yourself!
TMC: Teaching and Mentoring Communities, Inc
